# Supplementary material for: ARL5b inhibits human rhinovirus 16 propagation and impairs macrophage-mediated bacterial clearance
Source: EMBO Rep. 2024 Feb 8;25(3):16. doi: 10.1038/s44319-024-00069-x (PMC10933434; doi:10.1038/s44319-024-00069-x)
Supplement: Supplementary file 8 — Source Data Fig. 5 [file 44319_2024_69_MOESM8_ESM.zip › Figure 5/5A/READ ME 5A.docx]

Images were opened with ImageJ and cropped to show only the band corresponding to the protein of interest. Brightness and contrast were minimally adjusted (equally across the entire image) to show all relevant bands.
