# Supplementary figures and images for: ARL5b inhibits human rhinovirus 16 propagation and impairs macrophage-mediated bacterial clearance
Source: EMBO Rep. 2024 Feb 8;25(3):16. doi: 10.1038/s44319-024-00069-x (PMC10933434; doi:10.1038/s44319-024-00069-x)

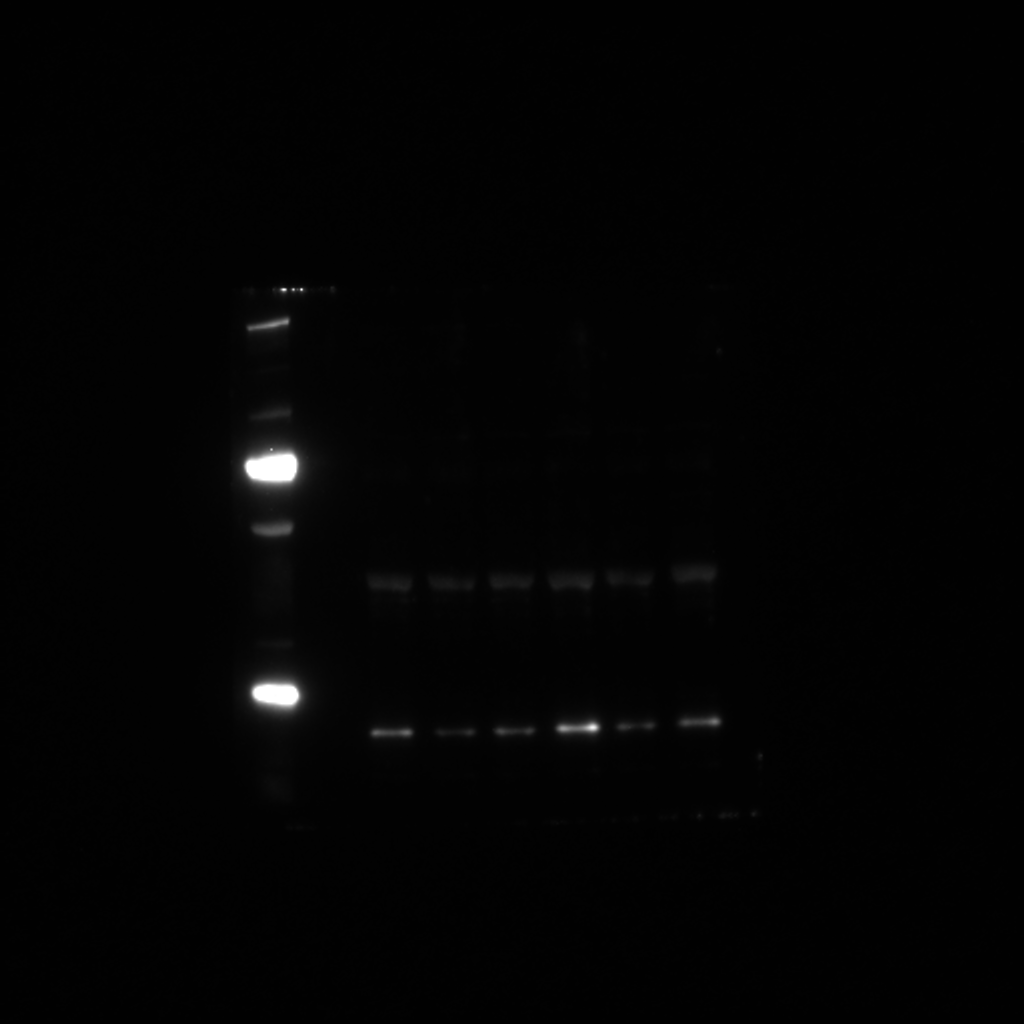

Supplement: Supplementary file 8 — Source Data Fig. 5 [file 44319_2024_69_MOESM8_ESM.zip › Figure 5/5A/ARL5b/ARL5b.Tif]

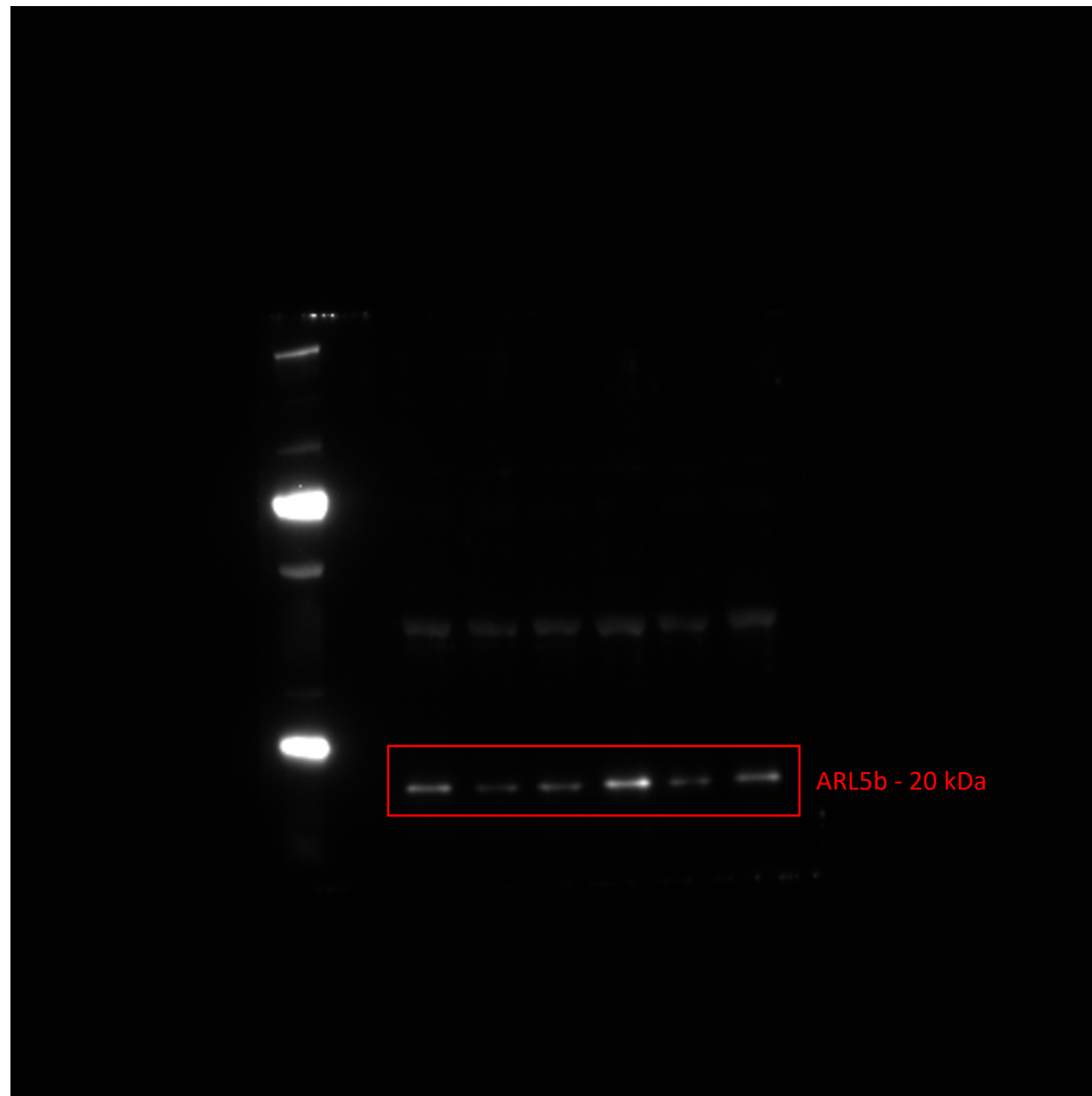

Supplement: Supplementary file 8 — Source Data Fig. 5 [file 44319_2024_69_MOESM8_ESM.zip › Figure 5/5A/ARL5b/ARL5b-annotated.pdf]

## Slide 1
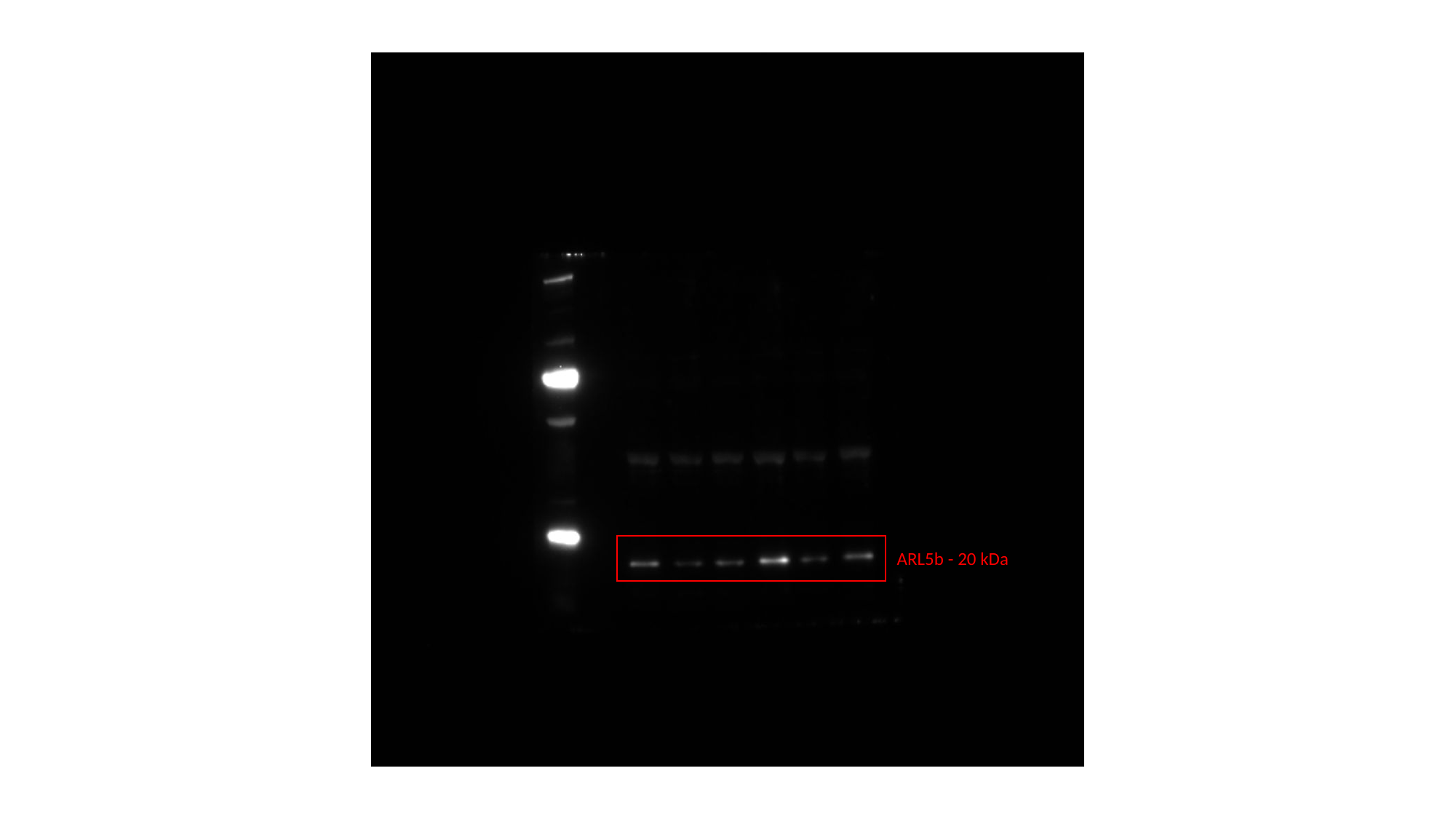

ARL5b - 20 kDa

Supplement: Supplementary file 8 — Source Data Fig. 5 [file 44319_2024_69_MOESM8_ESM.zip › Figure 5/5A/ARL5b/ARL5b-annotated.pptx]

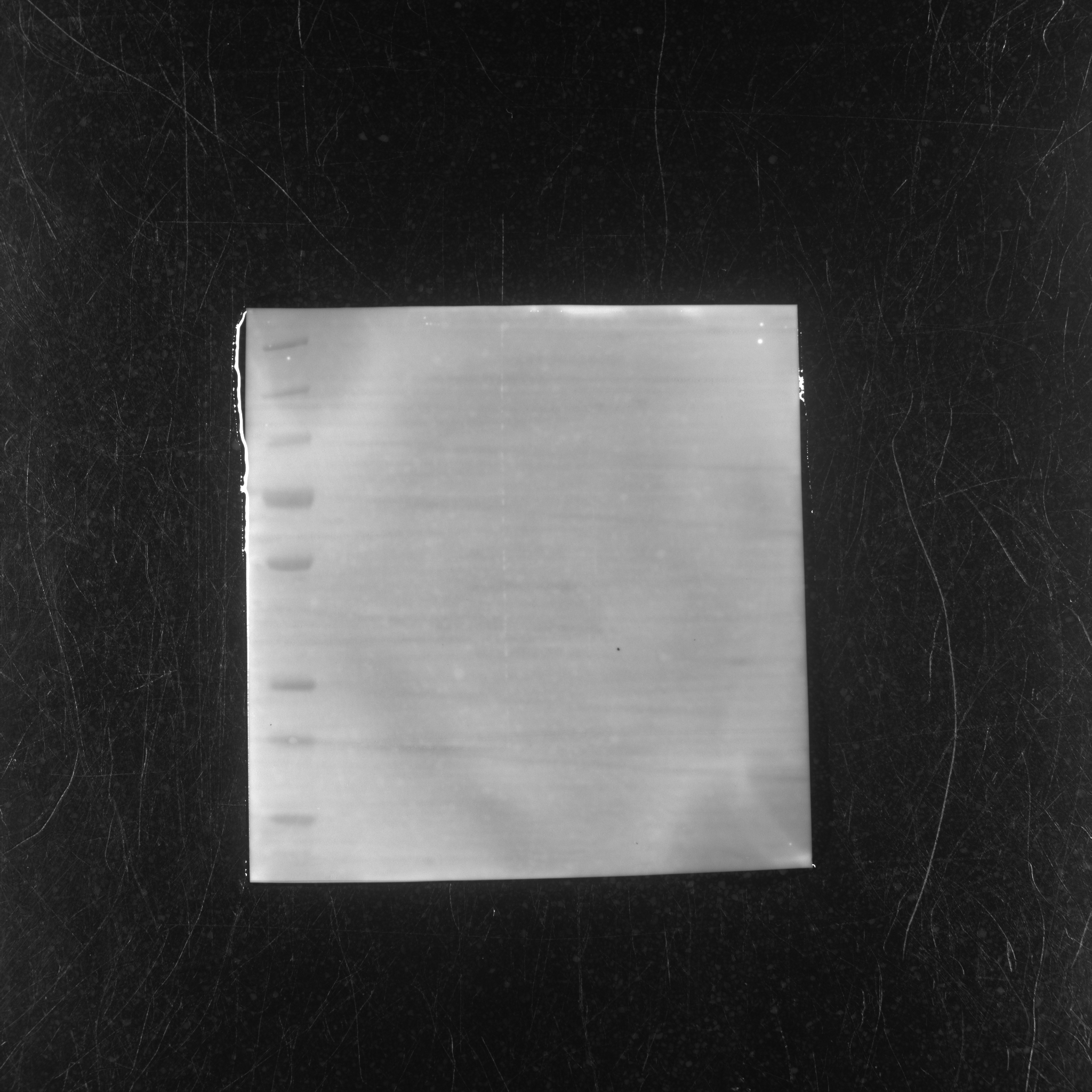

Supplement: Supplementary file 8 — Source Data Fig. 5 [file 44319_2024_69_MOESM8_ESM.zip › Figure 5/5A/ARL5b/ARL5b-ladder.Tif]

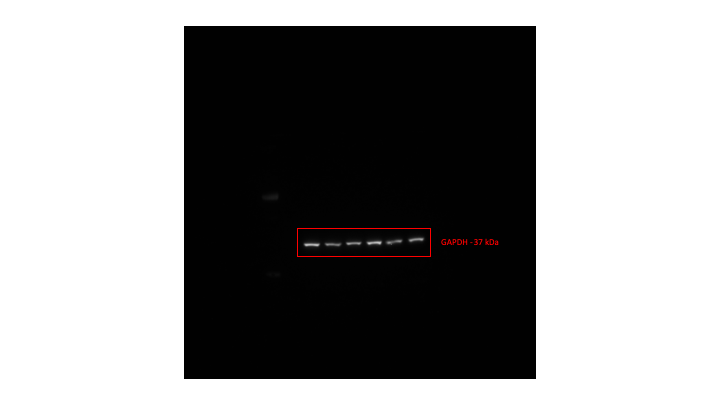

Supplement: Supplementary file 8 — Source Data Fig. 5 [file 44319_2024_69_MOESM8_ESM.zip › Figure 5/5A/GAPDH/GAPDH-annotated.tiff]

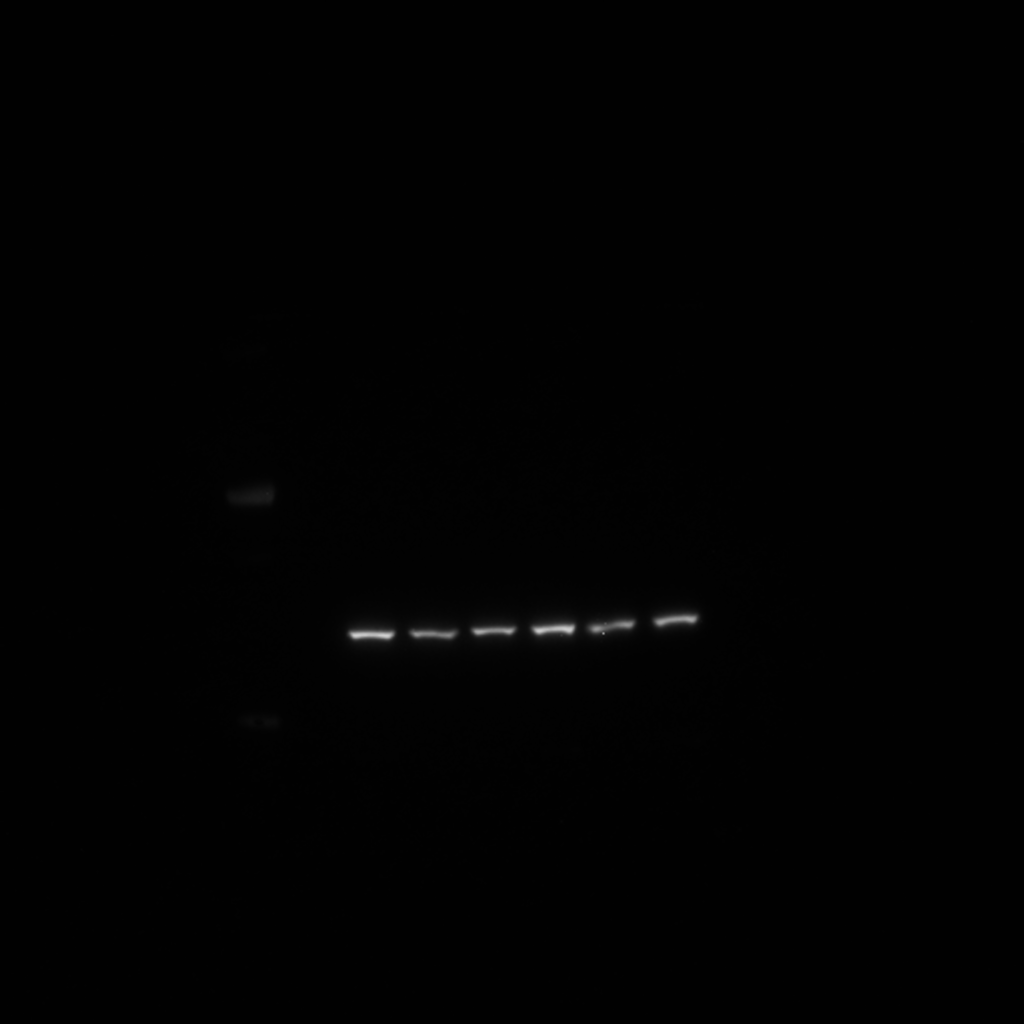

Supplement: Supplementary file 8 — Source Data Fig. 5 [file 44319_2024_69_MOESM8_ESM.zip › Figure 5/5A/GAPDH/GAPDH.Tif]

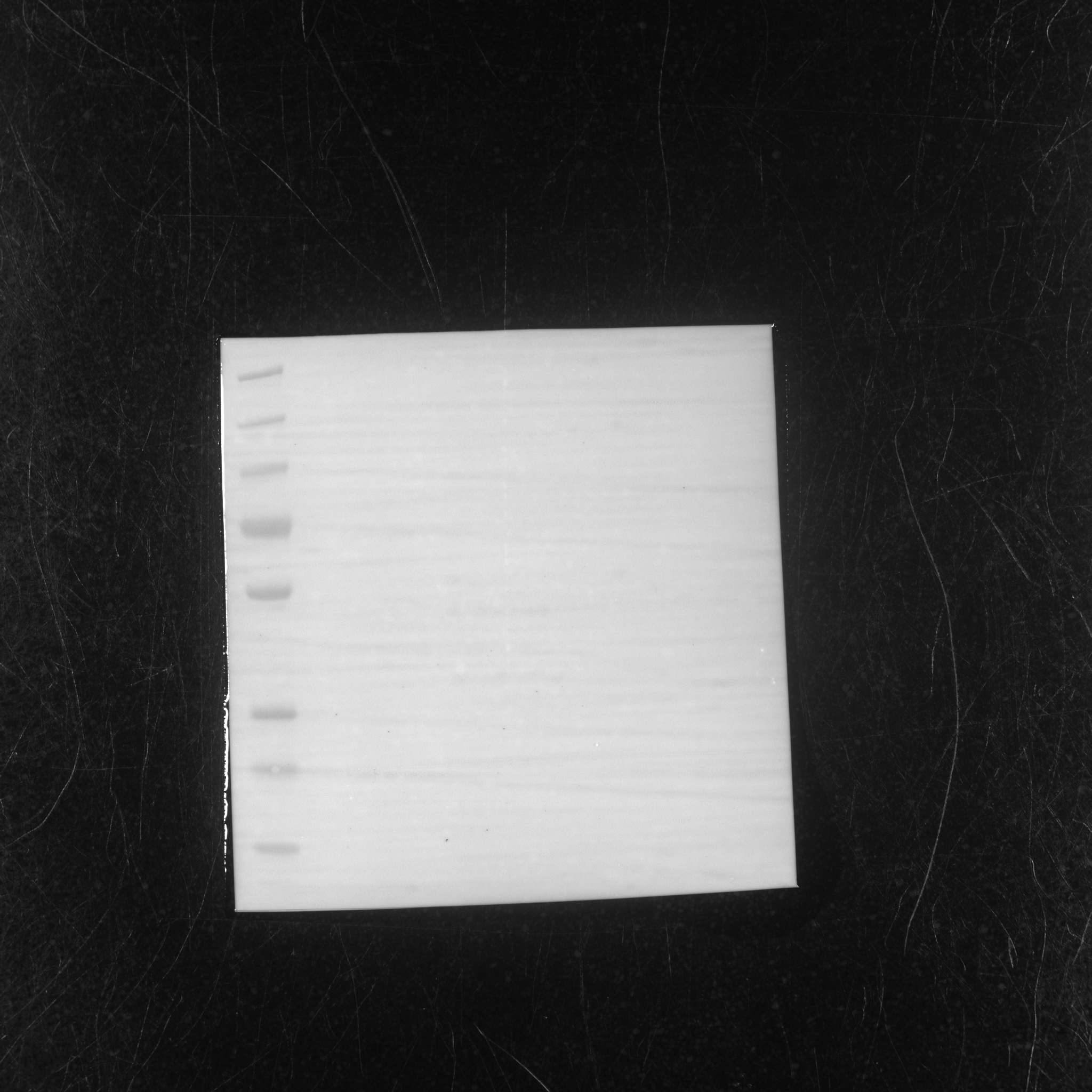

Supplement: Supplementary file 8 — Source Data Fig. 5 [file 44319_2024_69_MOESM8_ESM.zip › Figure 5/5A/GAPDH/GAPDH-ladder.Tif]

## Slide 1
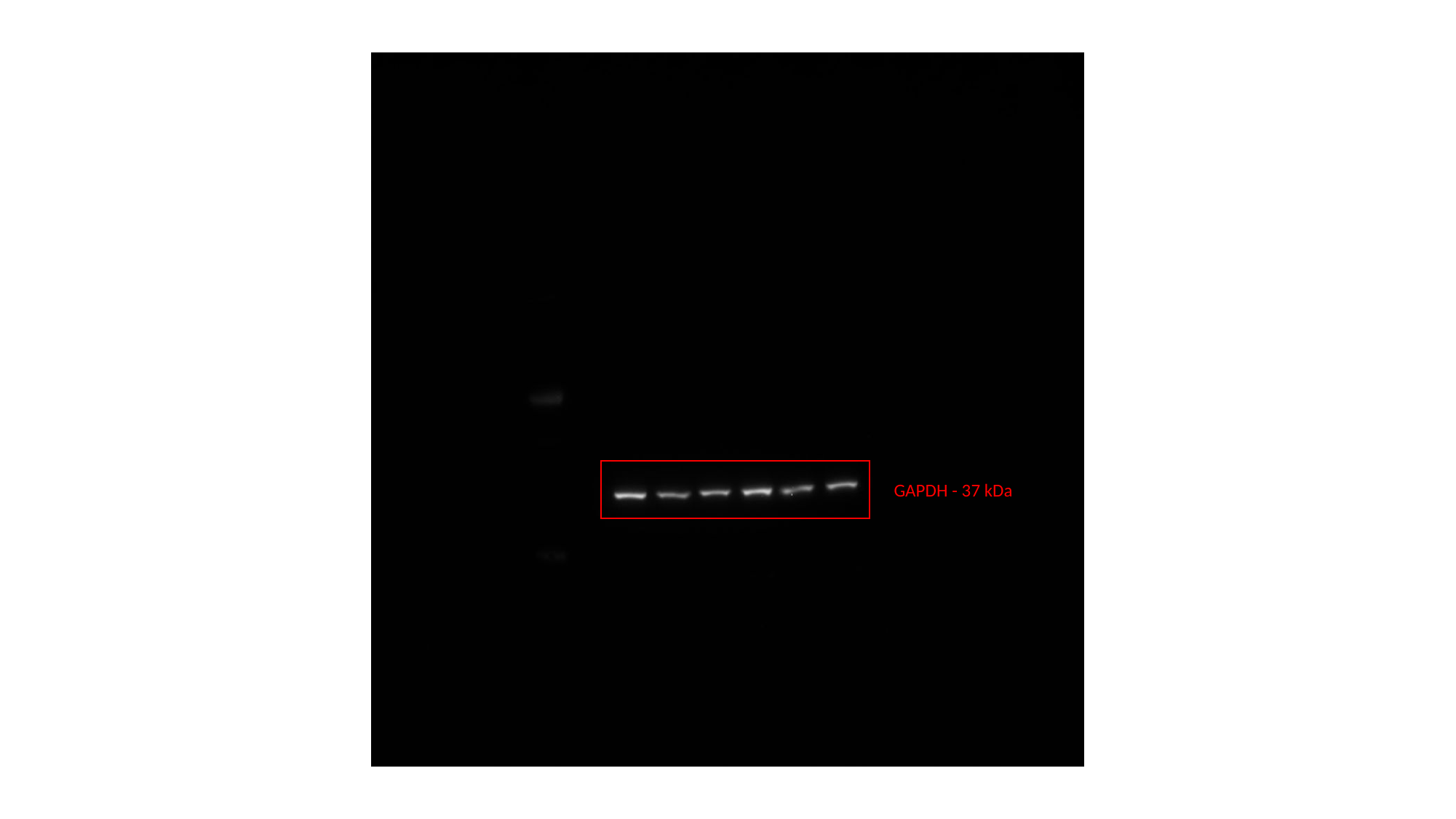

GAPDH - 37 kDa

Supplement: Supplementary file 8 — Source Data Fig. 5 [file 44319_2024_69_MOESM8_ESM.zip › Figure 5/5A/GAPDH/GAPDH-annotated.pptx]
