# Supplementary material for: ARL5b inhibits human rhinovirus 16 propagation and impairs macrophage-mediated bacterial clearance
Source: EMBO Rep. 2024 Feb 8;25(3):16. doi: 10.1038/s44319-024-00069-x (PMC10933434; doi:10.1038/s44319-024-00069-x)
Supplement: Supplementary file 11 — Expanded View Figures [file 44319_2024_69_MOESM11_ESM.pdf]

Expanded View Figures

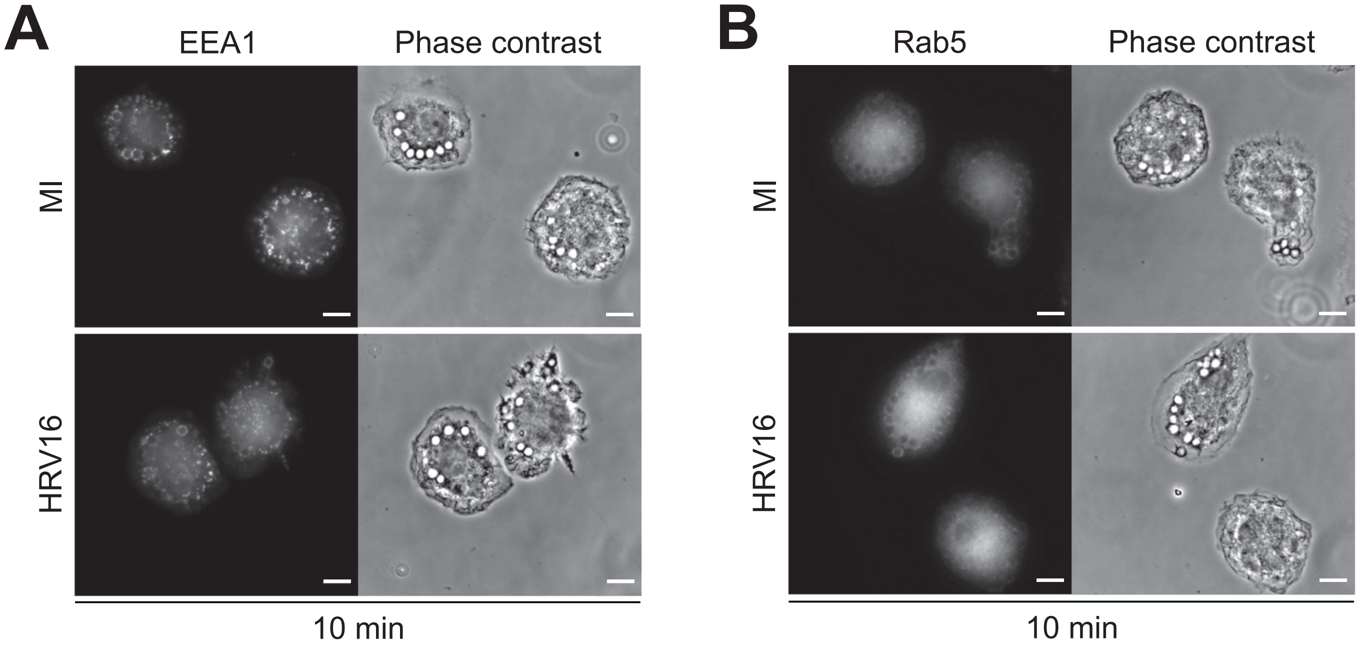

Figure EV1. HRV16 does not impair EEA1 or Rab5 recruitment to phagosomes after 10 min.

(**A**, **B**) hMDMs were challenged with HRV16 or MI and then exposed to IgG-opsonized sheep red blood cells for 10 min and either stained for (**A**) EEA1 or (**B**) Rab5. Representative images of (**A**) EEA1 or (**B**) Rab5 staining for MI (upper row) and HRV16-treated cells (lower row) are shown. Data information: (**A**, **B**) Scale bar represents 10 μm.

 © The Author(s)

A

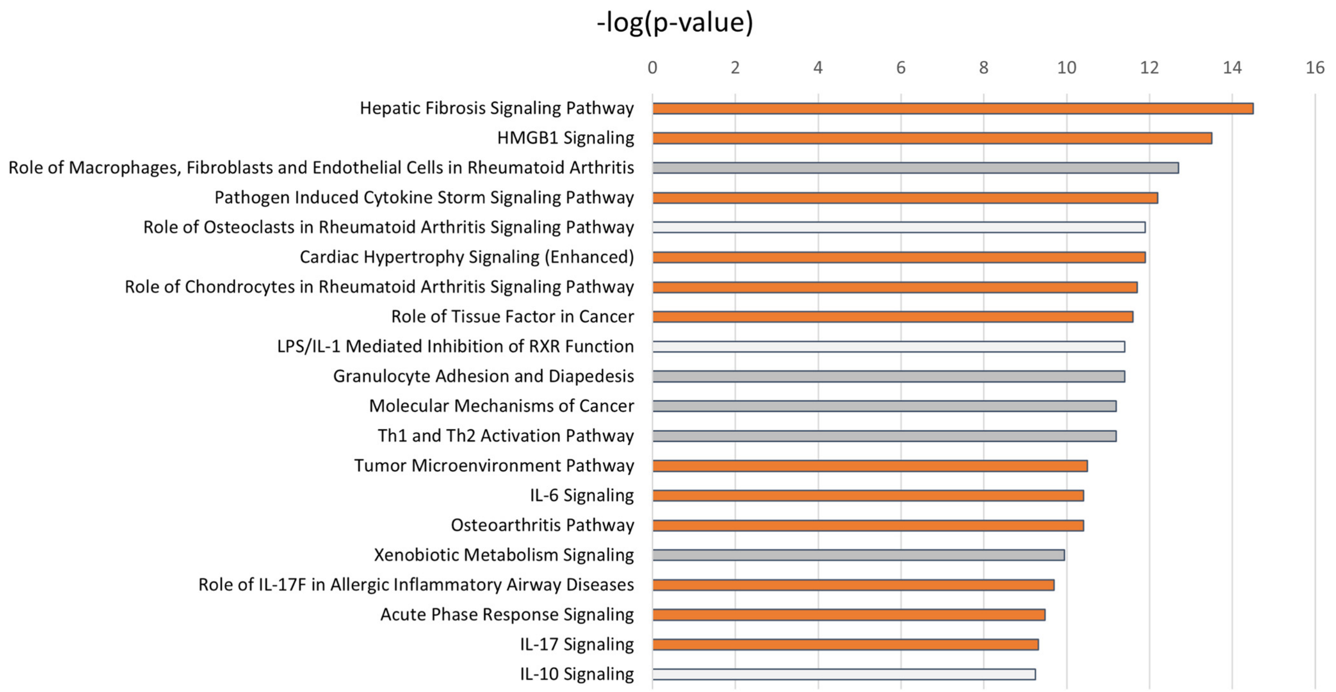

B

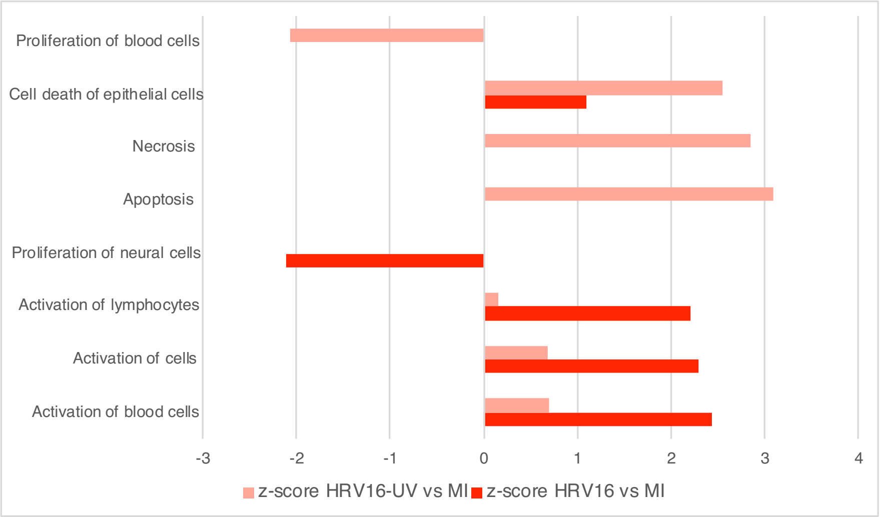

Figure EV2. Transcriptomic analysis of HRV16-treated macrophages.

hMDMs were challenged with HRV16, HRV16^{UV} or MI and characterized by RNA sequencing, $n = 6$. Data were analyzed using Ingenuity Pathway Analysis. (**A**) Most dysregulated pathways in HRV16-treated cells compared to MI. Orange bars indicate a predicted significantly activated pathway with a z-score >2. White bars indicate a predicted non-significantly activated pathway with a −2>z-score >2. Gray bars indicate that the pathway does not have a directionality that allow activation/inhibition to be predicted. (**B**) Dysregulated pathways in the HRV16-treated or HRV16^{UV}-treated cells compared to MI as determined from the 160 genes showing a larger variation than the normal spread when comparing the fold changes induced by HRV16 or HRV16^{UV}. Data information: *P* values and z-scores were evaluated with Ingenuity Pathway Analysis. Right-tailed Fisher's exact test statistical analysis was performed.

© The Author(s)

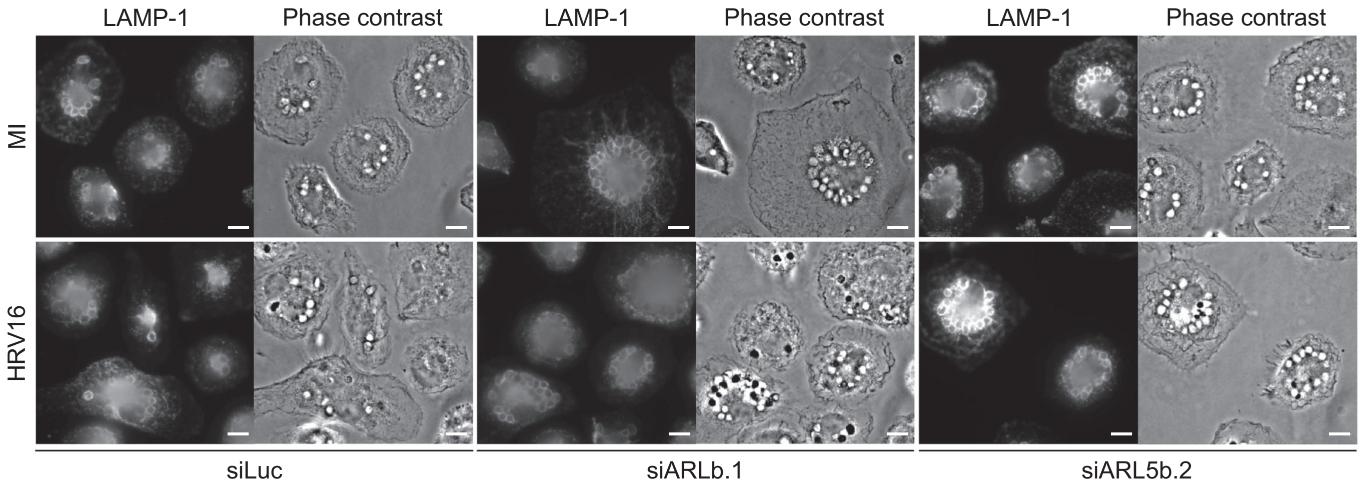

Figure EV3. ARL5b depletion restores the recruitment of LAMP-1 to phagosomes in HRV16-treated macrophages.

hMDMs were transfected with siRNA against luciferase (siLuc, control) or ARL5b (siARL5b.1 and siARL5b.2) and challenged with HRV16 or MI. Cells were exposed to IgG-opsonized sheep red blood cells for 60 min and LAMP-1 was stained. Representative images of LAMP-1 staining for MI (upper row) and HRV16-treated cells (lower row) for each siRNA are shown. Data information: Scale bar represents 10 µm.

 © The Author(s)
